# Amputation Case

**Published:** 1855-07

**Authors:** 


*Amputation Case.*

[The following letters sufficiently explain themselves. We published the article of Dr. Higgins in the March No. of the Journal, because it was requested by the Lasalle Co. Medical Society. We now publish the letters as an act of justice and fairness to the other side. Our only object is, to do justice to all the parties, who are personally strangers to us.]—ED.

---

TRENTON, Lasalle Co., Ill.,

May 25, 1855.

To the Editors of the North-Western Med. and Surg. Journal:

GENTLEMEN,—In the March No. of your Journal, my attention was attracted to an article entitled "Amputation," signed J. Higgins, in which there is an evident design, at least it so appears to me, knowing as I do all the antecedents of both parties, to gratify petty jealousies and private spleen, and not as should be and I might say would be the case with an honorable man, professional or otherwise, for the benefit of science.

I am aware it is difficult for all to know the motives which actuate good men, much more bad ones; this probably may account in some degree for the publication of the article in your popular Journal. The writer is fully aware of the object of the Journal, and therefore begs leave to correct any erroneous impressions which may have been made by your admitting the statement of a few facts.

In the fall of 1852, at the request of the same J. Higgins, and upon representations made by him to me (which representations proved to be untrue), I succeeded in obtaining the reluctant consent of the same Doc. ——— spoken of in the amputation article, to enter into copartnership with him. Dr. Weeks, shortly after the union was effected, left for New York city to spend the winter in the hospitals; on his return in the spring, finding that he had been deceived, censured me not a little for misrepresenting J Higgins and his professional business. I undertook to apologize for J. Higgins, but this would not satisfy Dr. Weeks, who told me he could readily submit to pecuniary loss, but that he would not be associated with one whose conduct was as unprofessional and dishonorable as J. Higgins. He consequently compelled the said Higgins to dissolve the co-partnership. This last act of Dr. Weeks, together with his general good success, is the inception of the "Amputation" article. I send you with this a statement of the case certified to by Dr. Winslow. Yours truly,

C. TODD.

---

PERU, May 20th, 1855.

CYRUS TODD, Esq., Trenton.

DEAR SIR,—As you have read the article in the Chicago Medical and Surgical Journal, of March, signed "John Higgins, M.D.," and wish a statement of the facts in the case, I will give them as near as I recollect.

July 22, 1854, A. Odekirk, of Trenton, was injured in this city by a train of cars passing over the left leg, just above the ankle, as it laid on the rail. I was called soon after and found the leg completely crushed. I called to my aid, Dr. E. Winslow, (who Dr. Higgins says is the best surgeon in town). As amputation was the only thing to be done, we did that in the usual manner (for circular operation) only using the saw for beveling the anterior surface of the tibia, where the forceps are sometimes used for the same purpose. As the article states, an artery spirted blood in my face, I do not recollect—I *have* sometimes got bloody in operations. As to the length of time consumed in performing the operation, I do not know what it was, perhaps half an hour; one thing is certain, it was not hurried, and I have been taught that an operation "was done quick enough when done well." As to the protrusion of the bone, I have never heard of any such occurrence, only through the channel of the article alluded to. But as you know the author, and the probable motive for his appearance in print, I think a longer notice superfluous.

I remain, most respectfully your friend,

J. F. WEEKS.

---

The above is a correct statement of the case.

E. WINSLOW.

